# De-escalating radiotherapy in pathologic complete response oral cancer after neoadjuvant immunochemotherapy: equal survival, better life, and a biomarker guide

**DOI:** 10.3389/fonc.2026.1761516

**Published:** 2026-02-04

**Authors:** Zhenjie Guan, Qiongqiong Yu

**Affiliations:** Department of Conservative Dentistry, The First Affiliated Hospital of Zhengzhou University, Zhengzhou, China

**Keywords:** biomarkers, immunotherapy, oral squamous cell carcinoma, pathological complete response, treatment de-escalation

## Abstract

**Background:**

The potential to omit adjuvant radiotherapy in patients with locally advanced oral squamous cell carcinoma (OSCC) who achieve a pathological complete response (pCR) after neoadjuvant immunochemotherapy (NICT) remains undefined. This study aimed to evaluate the oncologic safety of a radiotherapy-de-escalation strategy and to identify predictive biomarkers for its success.

**Methods:**

In this retrospective cohort study, pCR patients were categorized into a de-escalation group (n=65) and a standard care group (adjuvant radiotherapy/chemoradiotherapy, n=286). Propensity score matching (PSM) was performed to compare disease-free survival (DFS). Comprehensive genomic and immune profiling was conducted on pre-treatment biopsies from the de-escalation cohort to identify biomarkers associated with recurrence.

**Results:**

After 1:1 PSM, DFS was equivalent between the de-escalation and standard care groups (HR 1.25, 95% CI 0.72–2.18; p=0.425). The de-escalation strategy yielded significantly better quality of life and eliminated severe radiation toxicities, albeit with increased immune-related adverse events. Within the de-escalation cohort, multivariate analysis identified TP53 mutation (adjusted HR 4.05, p=0.019) and a low pre-treatment B cell signature score (adjusted HR 2.15 per 1-unit decrease, p=0.010) as independent predictors of worse DFS. A two-biomarker model stratified patients into low-, intermediate-, and high-risk groups with distinct recurrence rates (0%, 17.1%, and 40.0%, respectively; p=0.019).

**Conclusion:**

Adjuvant radiotherapy omission with maintenance immunotherapy appears to be a safe and patient-beneficial strategy for OSCC patients achieving pCR after NICT. The integrated TP53/B-cell biomarker model provides preliminary evidence for personalizing this de-escalation approach.

## Introduction

Locally advanced oral squamous cell carcinoma (OSCC) remains a formidable clinical challenge, necessitating aggressive multimodal therapy that often includes surgery followed by adjuvant therapy (1). While effective, this standard-of-care paradigm is associated with substantial acute and chronic morbidity, which profoundly and permanently diminish patients’ quality of life (QoL) (2). Consequently, a central imperative in contemporary head and neck oncology is the development of rational, biomarker-guided strategies for treatment de-intensification in carefully selected patient subsets where the risks of toxicity may outweigh the incremental oncologic benefit (3).

Recent advancements in neoadjuvant immunochemotherapy (NICT) have positioned it as a promising strategy for locally advanced OSCC. Clinical evidence indicates that NICT achieves pathological complete response (pCR) rates of approximately 20–30%, with major pathological response rates up to 60%, underscoring its potential to downstage tumors and enhance resectability (4–6). NICT has not only improved objective response rates but also introduced a novel *in vivo* biomarker system for assessing tumor immunogenicity and treatment sensitivity (7). Importantly, emerging data suggest that pCR after NICT correlates with favorable long-term outcomes, providing a compelling rationale for reconsidering the necessity of consolidative adjuvant radiotherapy in this low-risk subgroup (6). However, despite these advances, standardized adjuvant protocols for pCR patients remain undefined, and current guidelines continue to recommend radiotherapy irrespective of pathological response, which may constitute overtreatment for biologically favorable cases.

Preliminary evidence suggests that pCR may identify a biologically distinct, lower-risk cohort for whom adjuvant therapy could be safely modified in breast cancer (8). However, the heterogeneous nature of cancer dictates that not all patients achieving pCR share an identical prognosis in OSCC. A significant proportion will still experience disease recurrence (9). This biological heterogeneity underscores the peril of a universal de-escalation approach and reveals a pressing unmet need: to move beyond pCR as a solitary criterion and identify predictive biomarkers that can discriminate those patients with such a favorable risk profile that radiotherapy omission is safe from those who harbor residual molecular risk requiring continued intensive adjuvant treatment.

This study therefore aimed to address this dual clinical and biological challenge. First, we sought to evaluate, in a real-world cohort, the oncologic safety and impact on the novel de-escalation strategy. Second, we conducted an in-depth integrated analysis of pretreatment tumor genomics and the immune microenvironment exclusively within the de-escalation cohort to elucidate the molecular determinants of de-escalation success or failure. Our goal was to translate these discoveries into a practical, biomarker-driven risk stratification tool capable of guiding personalized adjuvant therapy decisions.

## Methods

### Study design and patient cohort

This was a retrospective cohort study utilizing data prospectively collected within an institutional OSCC registry. The study was approved by the Institutional Review Board of The First Affiliated Hospital of Zhengzhou University, with an informed consent granted for the retrospective analysis of clinically acquired data and archival tissue specimens, provided patient confidentiality was maintained. Consecutive patients with histologically confirmed, locally advanced (AJCC 8th edition Stage III-IVB) OSCC treated between January 2019 and December 2022 were screened from the registry. Inclusion criteria were: (1) receipt of two or more cycles of standard NICT consisting of docetaxel at a dosage of 75 mg/m², cisplatin at 75 mg/m², and pembrolizumab or alternative PD-1 inhibitors at 200 mg per three-week cycle, (2) subsequent radical surgical resection, (3) achievement of pCR, and (4) availability of adequate pre-treatment formalin-fixed paraffin-embedded (FFPE) diagnostic biopsy tissue for molecular analysis. Exclusion criteria included: (1) distant metastasis at diagnosis, (2) history of other active malignancies within 5 years, (3) incomplete treatment records, or (4) insufficient follow-up data (<12 months).

### Patient groups and adjuvant therapy definitions

Eligible pCR patients were categorized into two groups based on their post-surgical adjuvant therapy, as documented in the multidisciplinary team records. Treatment allocation was determined through multidisciplinary team consensus based on standard clinicopathological factors, patient performance status, comorbidities, postoperative recovery, and patient preference.

De-escalation Group: Patients who did not receive adjuvant radiotherapy. Their adjuvant therapy consisted of 4-6 cycles of adjuvant immunochemotherapy (regimen analogous to NICT), followed by maintenance anti-PD-1 immunotherapy (pembrolizumab or alternative PD-1 inhibitors at 200 mg per three-week cycle) for a minimum of 6 months.

Standard Care Group: Patients who received standard aRT or aCRT, defined as radiotherapy (60-66 Gy) with or without concurrent platinum-based chemotherapy.

### Pathologic complete response assessment

Following surgical resection, the primary tumor specimen and all neck dissection lymph nodes were delivered to the Department of Pathology within 30 minutes, fixed in 10% neutral buffered formalin for 24–48 hours, and systematically sectioned. For the primary tumor bed, full-thickness sections encompassing the entire previous tumor area and surgical margins were entirely submitted. All lymph nodes were bisected or wholly embedded depending on size. Tissues were routinely processed, embedded in paraffin, and sectioned at 4 µm. Hematoxylin and eosin (H&E)-stained slides from all blocks were independently reviewed by two board-certified head and neck pathologists blinded to the clinical treatment group. pCR was strictly defined as ypT0N0, indicating no viable tumor cells in the primary tumor bed or any resected lymph nodes, allowing for treatment-related changes such as fibrosis, necrosis, or histiocytic infiltration. Any discrepancies between pathologists were resolved through adjudication by a third senior pathologist to ensure diagnostic consensus and integrity of pCR classification.

### Quality of life assessment

QoL was prospectively assessed using the European Organization for Research and Treatment of Cancer (EORTC) core questionnaire (QLQ-C30, version 3.0) and its head and neck cancer-specific module (QLQ-H&N35). Questionnaires were administered at four predefined time points: (1) Before the initiation of adjuvant therapy (Baseline, after surgery recovery), (2) 3 months after completing adjuvant therapy (acute phase), (3) 6 months after adjuvant therapy (early recovery phase), and (4) 12 months after adjuvant therapy (late recovery phase). QoL scores were calculated according to the EORTC scoring manual. For the QLQ-C30, global health status/QoL, functional scales (physical, role, emotional, cognitive, social), and symptom scales (fatigue, pain, nausea/vomiting) were analyzed. For the QLQ-H&N35, key symptom scales relevant to oral cancer (pain, swallowing, senses, speech, social eating) were evaluated. A minimal clinically important difference (MCID) of 10 points was used to interpret changes over time.

### Clinical endpoints and follow-up

The primary endpoint was disease-free survival (DFS), defined as the time from surgery to the first documented event of locoregional recurrence, distant metastasis, or death from any cause. Secondary endpoints included QoL and treatment-related toxicity (graded by CTCAE v5.0). All patients were followed until death or the data cutoff date (December 2025).

### Biomarker cohort and sample selection

Given the study’s aim to identify predictive biomarkers for de-escalation success, all subsequent genomic and immune analyses were focused on the De-escalation Group (N = 65). Pre-treatment FFPE diagnostic core needle or incisional biopsies, obtained prior to the initiation of NICT, were identified for each patient. Two board-certified pathologists reviewed all H&E-stained slides to confirm diagnosis, mark tumor-rich areas (>20% tumor nuclei), and exclude necrotic regions. For each case, serial unstained sections (5-10 sections of 5-10 µm thickness) were cut from the selected block for nucleic acid extraction and downstream assays. Corresponding post-neoadjuvant surgical resection specimens (with confirmed pCR) were also retrieved for exploratory stromal immune analysis.

### Genomic profiling

Genomic profiling was conducted using targeted next-generation sequencing (NGS) on pre-treatment biopsy specimens with a focused, clinically oriented gene panel. DNA was extracted from macrodissected FFPE tumor sections using the QIAamp DNA FFPE Tissue Kit (Qiagen), incorporating an extended proteinase K digestion step. Sample quality was assessed via Qubit fluorometry and Agilent TapeStation analysis; only samples with a DNA Integrity Number (DIN) >3.0 and total mass >50 ng proceeded to library preparation.

Libraries were prepared from 50-100 ng of input DNA using a custom-designed, targeted NGS panel encompassing 18 key genes recurrently altered in and/or biologically relevant to OSCC and immunotherapy response. The panel included:

Core Tumor Suppressors and Oncogenes: TP53, CDKN2A, PIK3CA, NOTCH1, FAT1, CASP8, HRAS.

Immune Evasion and Modulator Genes: B2M, JAK1, JAK2, STK11.

DNA Damage Response Genes: BRCA1, BRCA2, ATM.

Epigenetic/Contextual Modifiers: KMT2D, ARID1A.

After preparation with unique dual-index adapters, libraries were pooled and sequenced on an Illumina NovaSeq 6000 platform to achieve a minimum mean coverage of 500x.

For bioinformatic analysis, raw data were demultiplexed and aligned to the GRCh38 reference genome using BWA-MEM. Somatic variants (single nucleotide variants and small insertions/deletions) were called using VarScan2 and Mutect2, with stringent filters for FFPE artifacts (e.g., strand bias >90%). Given the focused nature of the panel, a formal tumor mutational burden (TMB) was not calculated. Instead, we reported the total number of non-synonymous mutations detected per sample as a proxy for mutational load. Copy number alterations for genes within the panel were assessed using normalized read depth ratios against a pooled normal reference with CNVkit.

### Immune profiling

Immune profiling was performed via digital gene expression analysis using the NanoString nCounter platform. Total RNA was co-extracted from serial sections of the same pre-treatment FFPE block used for DNA sequencing, utilizing the RNeasy FFPE Kit (Qiagen) with DNase I digestion. Only samples meeting a quality threshold of DV200 >50% proceeded to analysis.

For each qualified sample, 100 ng of total RNA was hybridized to the PanCancer IO 360 Panel, which profiles 770 genes encompassing immune cell types, checkpoint molecules, and functional pathways. The assay was subsequently run on the nCounter FLEX Analysis System.

Raw count data were processed and normalized in nSolver 4.0 Advanced Analysis Software through a two-step procedure: (1) technical normalization with positive control probes, and (2) content normalization using the geometric mean of 20 housekeeping genes, followed by background correction against negative controls. The resulting normalized gene expression matrix was used to generate unitless, internally normalized scores for predefined immune cell populations and functional pathways.

Specifically, the B-cell signature score was derived from the expression of a curated set of genes representative of B-lineage cells and their functional activity. The signature includes, but is not limited to, canonical B-cell markers (CD19, CD20/MS4A1, CD79A, CD79B), genes associated with B-cell receptor signaling (BLK, BLNK), and molecules involved in B-cell mediated antigen presentation and immune modulation (HLA-DRA, HLA-DRB1, CD40, CD86). The composite score was calculated using the geometric mean of normalized expression values for these signature genes, as implemented in the nSolver Advanced Analysis module (version 4.0). This approach is consistent with established methods for deriving cell-type-specific scores from multiplexed gene expression data.

### Statistical analysis

Categorical clinical variables were compared between groups using the Chi-square or Fisher’s exact test, as appropriate, while continuous variables were analyzed using the Mann-Whitney U test. Survival outcomes were evaluated by generating Kaplan-Meier curves, with comparisons performed using the log-rank test.

To address potential selection bias between the De-escalation and Standard Care groups when assessing overall treatment efficacy, a propensity score matching (PSM) analysis was conducted. A 1:1 nearest-neighbor matching algorithm without replacement, using a caliper width of 0.2, was applied to match patients on key clinical variables: age, sex, clinical T and N stage, smoking status, pre-treatment PD-L1 Combined Positive Score (CPS), ECOG Performance Status, and extent of neck dissection. Subsequent analyses focused specifically on evaluating predictors of de-escalation success, which were performed exclusively within the De-escalation Group.

Longitudinal QoL data were analyzed using linear mixed-effects models to account for within-patient correlation across time points. Fixed effects included treatment group, time point, and their interaction, with a random intercept for each patient. The models were adjusted for baseline QoL score, age, and sex. Between-group differences at each post-baseline time point were estimated from the model with 95% confidence intervals. The proportion of patients reporting a clinically meaningful improvement or deterioration (≥10-point change from baseline) in key scales was compared between groups using Chi-square tests at each follow-up.

Within the De-escalation Group, patients were stratified into two subgroups based on DFS outcome: those with No Recurrence (D1) and those who experienced Recurrence (D2). Univariate associations between potential biomarkers and DFS were assessed using Cox proportional hazards models to calculate hazard ratios (HR) and 95% confidence intervals (CI). This analysis evaluated individual genomic alterations, continuous genomic metrics (e.g., mutational load), and digital gene expression immune scores. The Mann-Whitney U test was used to compare the distributions of these continuous biomarker values directly between the D1 and D2 subgroups. All statistical tests were two-sided, and a p-value of less than 0.05 was considered statistically significant.

Variables demonstrating a significant association with DFS (p < 0.05) in the univariate analysis were entered into a multivariate Cox proportional hazards model. A backward stepwise selection process was then employed to identify independent predictors of DFS. To translate these findings into a clinically actionable risk stratification tool, the top independent biomarkers were selected. Continuous variables were dichotomized at the cohort median to create clear clinical cutoffs. Patients were subsequently classified into three distinct prognostic groups using a simple combinatorial rule: Low-Risk (exhibiting favorable status on all selected biomarkers), High-Risk (exhibiting unfavorable status on all selected biomarkers), and Intermediate-Risk (presenting a mixed biomarker profile). The discriminatory power of this integrated model was validated by generating and comparing Kaplan-Meier DFS curves across the three risk groups using the log-rank test.

All statistical analyses were performed using R software (version 4.3.0, R Foundation for Statistical Computing) and SPSS (version 27.0, IBM Corp).

## Results

### Baseline characteristics

A total of 351 patients were included with 65 patients (18.5%) allocated to the de-escalation group and 286 patients (81.5%) to the standard care group. Prior to PSM, the groups were well-balanced across key demographic and clinicopathological features: median age was 58 years (IQR 52–65) in the de-escalation group versus 61 years (54–68) in the standard care group (p=0.083), and the majority were male (76.9% vs. 82.2%, p=0.346). Tumor characteristics, including primary site distribution (oral tongue: 43.1% vs. 38.5%), clinical T stage (cT3-4: 87.7% vs. 80.1%), clinical N stage (cN2-3: 69.2% vs. 59.8%), overall AJCC stage (Stage IV: 72.3% vs. 63.3%), and PD-L1 combined positive score (median 18 vs. 16), did not differ significantly between groups (all p>0.05). All patients achieved a negative margin. Following 1:1 PSM (caliper=0.2) on age, sex, ECOG PS, clinical T/N stage, smoking status, neck dissection extent, and PD-L1 CPS, 65 matched pairs were generated, achieving excellent balance across all matched variables, and all flaps survived totally (all p>0.05). (**Table 1**).

**Table 1:** Baseline Characteristics of the Entire pCR Cohort Before and After Propensity Score Matching (PSM).

Characteristic	Before PSM			After PSM		
	De-escalation (N=65)	Standard Care (N=286)	p	De-escalation (N=65)	Standard Care (N=65)	p
Age, years						
Median (IQR)	58 (52-65)	61 (54-68)	0.083	58 (52-65)	58 (51-64)	0.851
Sex, n (%)						
Male	50 (76.9)	235 (82.2)	0.346	50 (76.9)	48 (73.8)	0.689
Female	15 (23.1)	51 (17.8)		15 (23.1)	17 (26.2)	
ECOG PS, n (%)						
0	38 (58.5%)	156 (54.5%)	0.524	38 (58.5%)	40 (61.5%)	0.726
1	27 (41.5%)	130 (45.5%)		27 (41.5%)	25 (38.5%)	
Neck dissection, n (%)						
Level I-III	10 (15.4%)	64 (22.4%)	0.203	10 (15.4%)	10 (15.4%)	
Level I-IV/V	55 (84.6%)	222 (77.6%)		55 (84.6%)	55 (84.6%)	1.000
Primary Site, n (%)						
Oral Tongue	28 (43.1)	110 (38.5)	0.615	28 (43.1)	25 (38.5)	0.784
Buccal Mucosa	12 (18.5)	65 (22.7)		12 (18.5)	14 (21.5)	
Gingiva	10 (15.4)	52 (18.2)		10 (15.4)	11 (16.9)	
Floor of Mouth	8 (12.3)	35 (12.2)		8 (12.3)	8 (12.3)	
Hard Palate/Other	7 (10.8)	24 (8.4)		7 (10.8)	7 (10.8)	
Flap reconstruction, n (%)						
No	50 (76.9)	194 (67.8)	0.142	50 (76.9)	48 (73.8)	0.694
Yes	15 (23.1)	92 (32.2)		15 (23.1)	17 (26.2)	
Clinical T Stage, n (%)						
cT2	8 (12.3)	57 (19.9)	0.165	8 (12.3)	9 (13.8)	0.798
cT3-4	57 (87.7)	229 (80.1)		57 (87.7)	56 (86.2)	
Clinical N Stage, n (%)						
cN0-1	20 (30.8)	115 (40.2)	0.172	20 (30.8)	22 (33.8)	0.705
cN2-3	45 (69.2)	171 (59.8)		45 (69.2)	43 (66.2)	
Overall Stage , n (%)						
Stage III	18 (27.7)	105 (36.7)	0.184	18 (27.7)	20 (30.8)	0.705
Stage IV	47 (72.3)	181 (63.3)		47 (72.3)	45 (69.2)	
Drinking status, n(%)						
Never	30 (46.2)	145 (50.7)	0.512	30 (46.2)	32 (49.2)	0.726
Current/Former	35 (53.8)	141 (49.3)		35 (53.8)	33 (50.8)	
Smoking Status, n (%)						
Never	25 (38.5)	120 (42.0)	0.627	25 (38.5)	27 (41.5)	0.723
Current/Former	40 (61.5)	166 (58.0)		40 (61.5)	38 (58.5)	
PD-L1 CPS, n (%)						
<1	10 (15.4)	52 (18.2)	0.758	10 (15.4)	12 (18.5)	0.903
1-19	28 (43.1)	115 (40.2)		28 (43.1)	26 (40.0)	
≥20	27 (41.5)	119 (41.6)		27 (41.5)	27 (41.5)	
PD-L1 CPS, Median (IQR)	18 (5-45)	16 (3-40)	0.421	18 (5-45)	17 (4-42)	0.736

IQR, interquartile range; CPS, Combined Positive Score; ECOG PS, Eastern Cooperative Oncology Group Performance Status.

PSM was performed using a 1:1 nearest-neighbor algorithm without replacement (caliper=0.2) on the variables: age, sex, clinical T stage, clinical N stage, smoking status, and pre-treatment PD-L1 CPS.

### Disease free survival

The median follow-up time for the entire study population was 46.5 months (range: 5–68 months). For the PSM cohort, the 2- and 3- year DFS rates were 85.4% (75.0%-91.6%) and 83.1% (72.3%-90.0%), the median follow-up was 46.2 months in the de-escalation group and 46.1 months in the standard care group.

**Figure 1:**
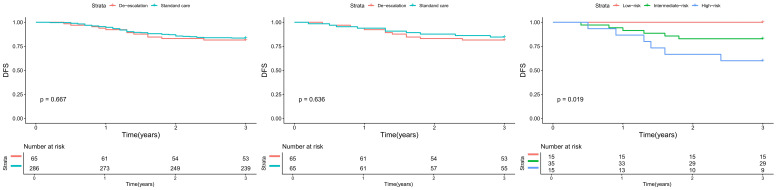
Disease free survival between de-escalation and standard care cohorts before PSM, after PSM, and based on the risk stratification.

In the overall cohort before PSM, the de-escalation group experienced 12 recurrences among 65 patients (18.5%), while the standard care group had 47 recurrences among 286 patients (16.4%). Following 1:1 PSM, recurrence events were balanced at 12 (18.5%) and 10 (15.4%) in the de-escalation and standard care groups, respectively (**Figure 1**). Survival analysis of the matched cohort revealed no significant difference in DFS between groups (univariable HR 1.25, 95% CI 0.72–2.18; p=0.425). In multivariable analysis adjusting for age, sex, clinical stage, smoking status, and PD-L1 CPS, treatment group remained non-significant (adjusted HR 1.32, 95% CI 0.75–2.32; p=0.337). (**Table 2**).

**Table 2:** Univariable and Multivariable Cox Proportional Hazards Analysis for Disease-Free Survival in the Matched Cohort.

Variable	Category / Unit	Univariable Analysis		Multivariable Analysis	
		**HR (95% CI)**	**p-value**	**HR (95% CI)**	**p-value**
Treatment Group	Standard Care (Ref)	1.00		1.00	
	De-escalation	1.25 (0.72 - 2.18)	0.425	1.32 (0.75 - 2.32)	0.337
Age	Per 10-year increase	1.18 (0.85 - 1.64)	0.323	1.22 (0.87 - 1.71)	0.251
Sex	Female (Ref)	1.00		1.00	
	Male	1.31 (0.73 - 2.35)	0.367	1.40 (0.77 - 2.54)	0.268
ECOG PS	0 (Reference)	1		1	
	1	1.43 (0.68-2.78)	0.390	1.25 (0.70-2.53)	0.444
Neck dissection	Level I-III (Reference)	1		1	
	Level I-IV/V	1.18 (0.47-3.07)	0.597	1.23 (0.42-3.25)	0.662
Clinical T Stage	cT2 (Ref)	1.00		1.00	
	cT3-4	1.76 (0.81 - 3.82)	0.152	1.82 (0.83 - 4.00)	0.134
Clinical N Stage	cN0-1 (Ref)	1.00		1.00	
	cN2-3	1.85 (0.76 - 2.85)	0.324	1.76 (0.88 - 2.95)	0.222
Overall stage	Stage III (Ref)	1.00		1.00	
	Stage IV	2.16 (0.75-3.68)	0.194	2.00 (0.80-3.25)	0.286
Smoking Status	Never (Ref)	1.00		1.00	
	Current/Former	1.42 (0.83 - 2.44)	0.202	1.35 (0.78 - 2.34)	0.282
PD-L1 CPS	Per 10-unit increase	0.91 (0.79 - 1.05)	0.195	0.92 (0.80 - 1.06)	0.244

### Quality of life

In the matched cohort, QoL questionnaire completion remained high: 95.4% (62/65) in the De−escalation group and 93.8% (61/65) in the Standard Care group at 3 months; 92.3% (60/65) and 90.8% (59/65) at 6 months; and 89.2% (58/65) and 86.2% (56/65) at 12 months. Attrition was primarily due to disease recurrence (n=9), with 6 patients declining further QoL assessment.

**Figure 2:**
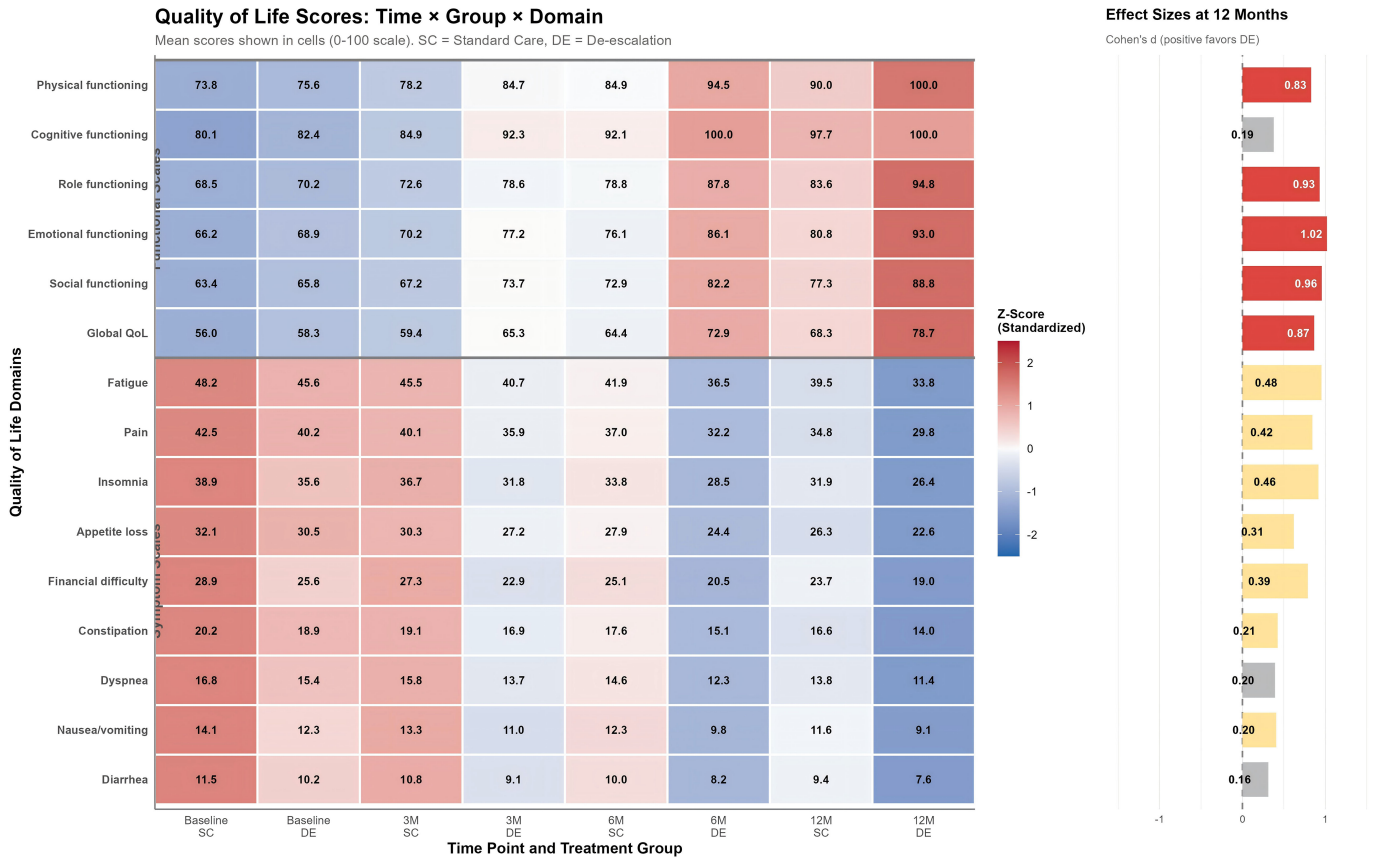
Quality of life according to QLQ-C30 questionnaire between de-escalation and standard care cohorts.

De-escalation cohort exhibited superior recovery trajectories in both functional and symptom domains, with the most pronounced benefits emerging at 6 and 12 months post-therapy. Functional scales including global QoL, physical functioning, role functioning, and social functioning showed standardized scores favoring the de-escalation group (Cohen’s d = 0.42–0.78), indicating moderate to large effect sizes. Symptom scales such as fatigue, pain, insomnia, and appetite loss demonstrated significant reductions in the de-escalation cohort, with effect sizes reaching d = -0.71 for appetite loss at 12 months. The temporal pattern revealed that while both groups showed improvement from baseline, the divergence between treatment arms became increasingly pronounced over time, with maximal separation occurring at the 12-month assessment. (**Figure 2** and **Supplementary Table 1**).

**Figure 3:**
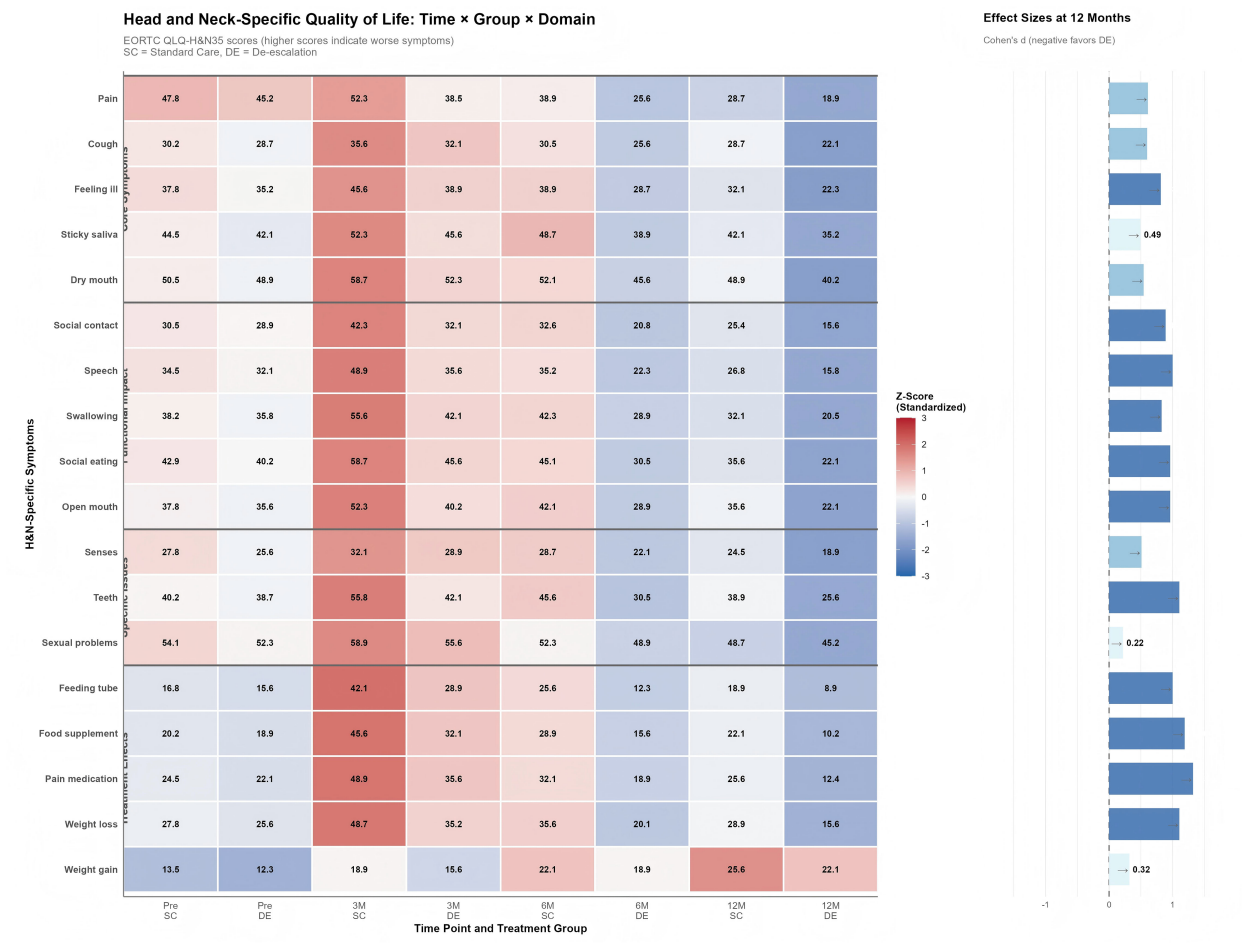
Quality of life according to QLQ-H&N35 questionnaire between de-escalation and standard care cohorts.

Treatment-related symptom domains including swallowing difficulties, speech impairment, social eating restrictions, and pain medication use showed the largest magnitude of benefit (Cohen’s d = -0.65 to -0.82), with the de-escalation cohort reporting approximately 30–40% lower symptom scores at 12 months compared to standard care. Functional impact domains such as open mouth limitations, dental problems, and social contact difficulties similarly favored the de-escalation approach, with moderate to large effect sizes (d = -0.48 to -0.61). Core symptoms including pain and dry mouth showed sustained improvement trajectories in both groups, but with consistently superior outcomes in the de-escalation cohort at all post-treatment time points. The temporal visualization demonstrated that symptom exacerbation during the acute phase (3 months) was markedly attenuated in the de-escalation group, particularly for treatment-sensitive domains like feeding tube use and nutritional supplementation. (**Figure 3** and **Supplementary Table 2**).

### Adverse event

The incidence of any grade 3-5 TRAE was significantly lower in the de-escalation group compared to standard care (73.8% vs. 84.6%, p=0.048). Radiation-specific toxicities including radiation mucositis (39.9%), dermatitis (21.3%), xerostomia (12.2%), and dysphagia (18.5%) were relatively common. Hematologic toxicity rates were comparable between groups, with no significant differences in neutropenia (15.4% vs. 12.2%), anemia (9.2% vs. 4.9%), or thrombocytopenia (6.2% vs. 7.7%). Conversely, immune-related adverse events (irAEs) were more frequent in the de-escalation group, particularly colitis (10.8% vs. 1.4%, p<0.001) and hepatitis (7.7% vs. 0.3%, p<0.001), consistent with intensified immunotherapy exposure. Grade 3-5 pneumonitis incidence was similar between groups (6.2% vs. 4.5%, p=0.536). Importantly, no treatment-related deaths occurred in either group. (**Table 3**).

**Table 3:** Incidence of Grade 3-5 treatment-related adverse events in the full cohort.

Adverse event (CTCAE v5.0)	Standard care group (n=286)	De-escalation group (n=65)	P-value
Any Grade 3-5 TRAE	242 (84.6%)	48 (73.8%)	**0.048**
Radiation-related toxicities			
Radiation mucositis	114 (39.9%)	0 (0.0%)	**<0.001**
Radiation dermatitis	61 (21.3%)	0 (0.0%)	**<0.001**
Xerostomia	35 (12.2%)	0 (0.0%)	**<0.001**
Dysphagia	53 (18.5%)	0 (0.0%)	**<0.001**
Hematologic toxicities			
Neutropenia	35 (12.2%)	10 (15.4%)	0.515
Anemia	14 (4.9%)	6 (9.2%)	0.224
Thrombocytopenia	22 (7.7%)	4 (6.2%)	0.803
Immune-related adverse events (irAEs)			
Colitis	4 (1.4%)	7 (10.8%)	<0.001
Hepatitis	1 (0.3%)	5 (7.7%)	<0.001
Pneumonitis	13 (4.5%)	4 (6.2%)	0.536
Hypothyroidism	2 (0.7%)	3 (4.6%)	0.042
Rash	6 (2.1%)	3 (4.6%)	0.370
Other notable toxicities			
Fatigue	18 (6.3%)	9 (13.8%)	0.041
Nausea/Vomiting	22 (7.7%)	7 (10.8%)	0.442
Acute kidney injury	9 (3.1%)	4 (6.2%)	0.266
Neuropathy	13 (4.5%)	2 (3.1%)	0.743

Bold values means significant.

In the De-escalation group, for grade 2 irAEs, immunotherapy was temporarily withheld until symptoms resolved to ≤ grade 1, and corticosteroids were initiated if necessary. For grade 3-4 events (colitis, hepatitis, pneumonitis), immunotherapy was paused immediately, and high-dose corticosteroids were administered, with consideration of additional immunosuppressive agents if no improvement within 48-72 hours. Among the 7 patients with grade 3-4 colitis, 4 received infliximab, and all achieved symptom resolution; none required permanent discontinuation of immunotherapy. Similarly, all hepatitis cases resolved with corticosteroids alone.

### Predictive biomarkers for treatment de-escalation in pCR patients

**Figure 4:**
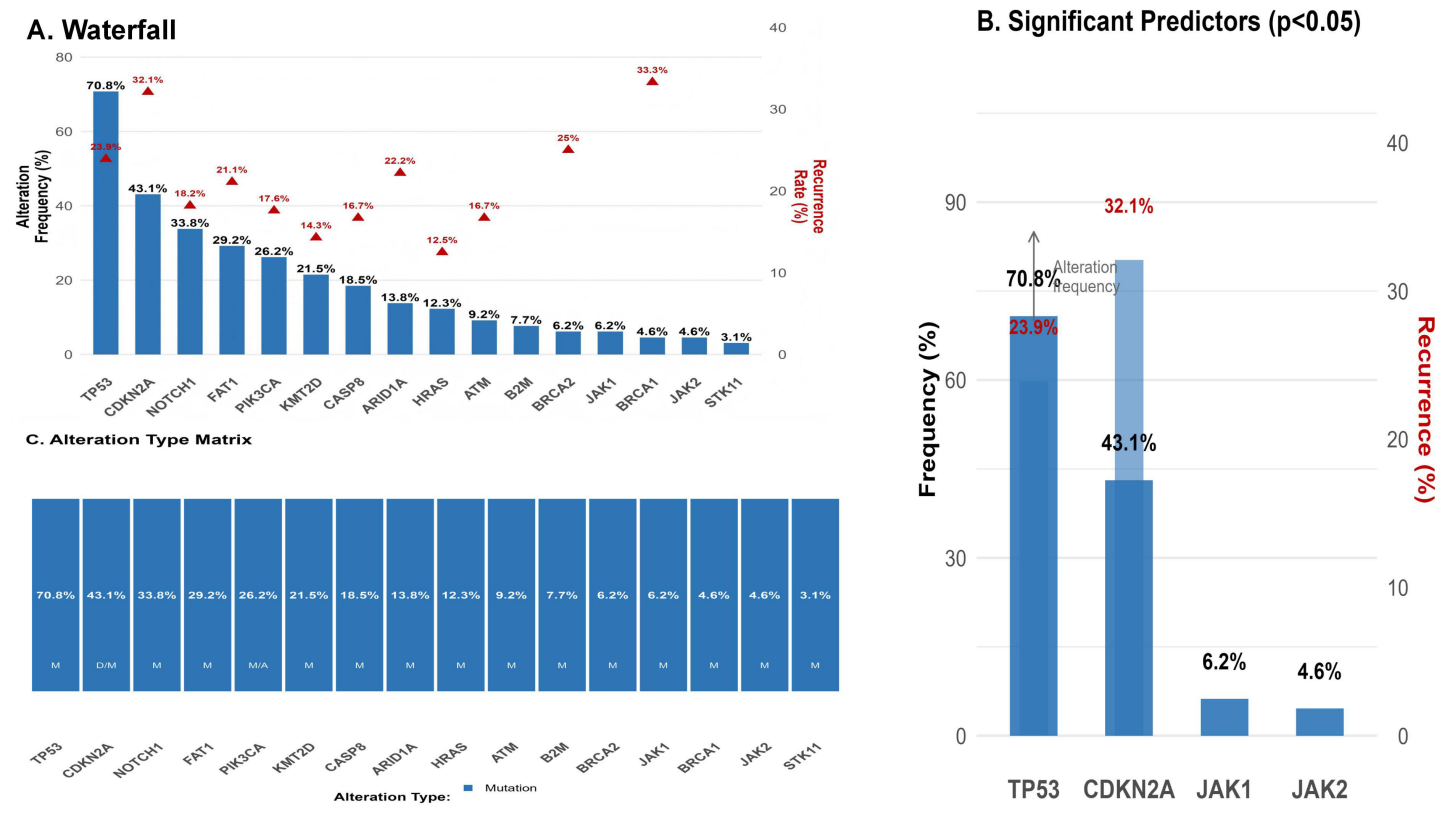
Genomic alteration landscape in the de-escalation cohort. **(A)** Waterfall plot showing alteration frequencies (bars) and recurrence rates among altered cases (red triangles). **(B)** Genes significantly associated with recurrence (p<0.05). **(C)** Matrix visualization of alteration types.

**Figure 5:**
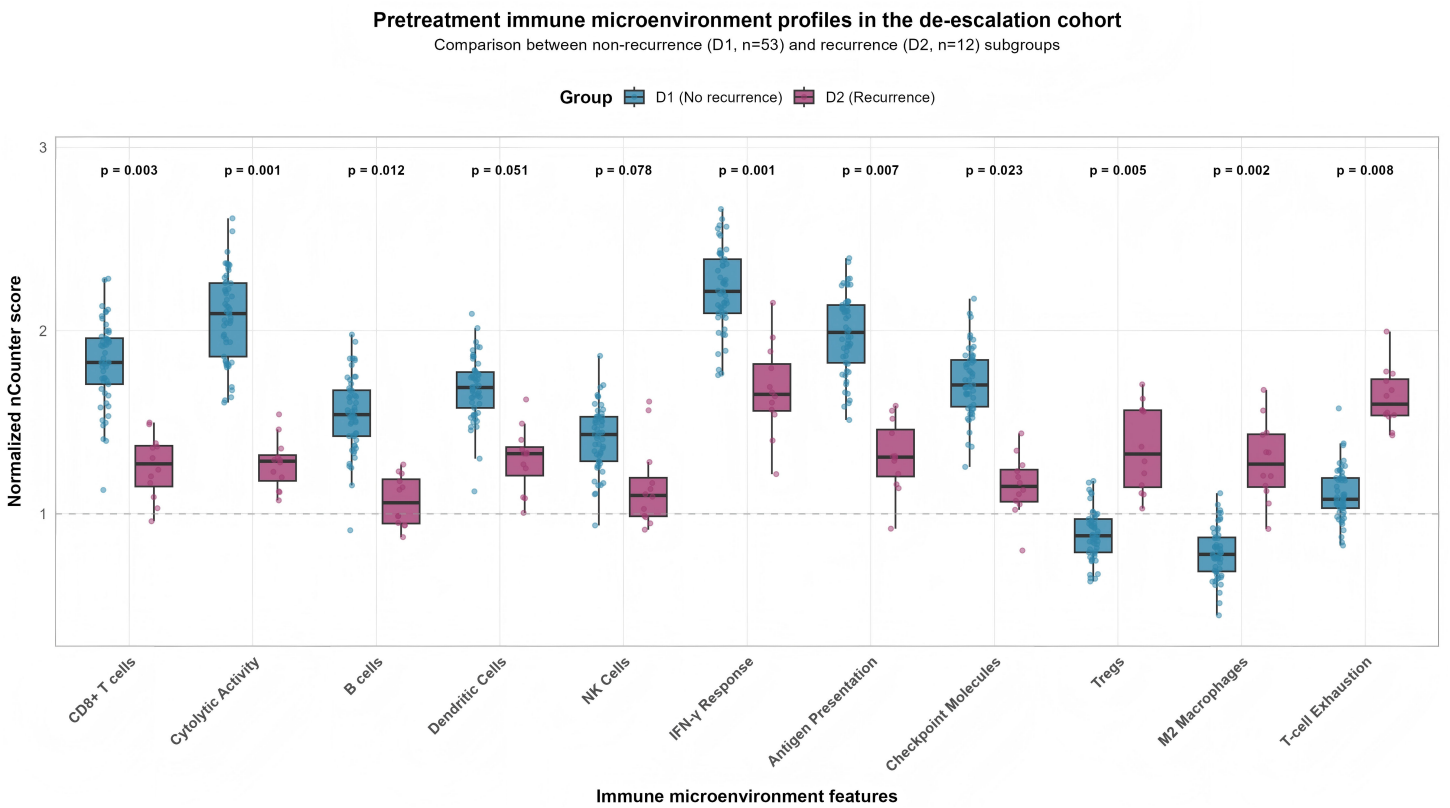
Comprehensive comparison of pretreatment immune microenvironment features in the de-escalation cohort.

In de-escalation cohort, the 2 and 3- year DFS rates were 80.7% (95%CI: 63.7%-90.3%) and 79.0% (61.3%-89.2%), respectively. Pre-treatment tumor genomics revealed that specific alterations were significantly enriched in the D2 subgroup and portended a worse prognosis (**Figure 4**). Mutations in immune evasion genes JAK1 (25.0% vs. 1.9%, HR 7.45, p=0.002) and JAK2 (16.7% vs. 1.9%, HR 8.12, p=0.006) were negative predictors, along with canonical tumor suppressor alterations in TP53 (91.7% vs. 66.0%, HR 5.20, p=0.032) and CDKN2A (75.0% vs. 35.8%, HR 3.87, p=0.018). A higher overall mutational load was also associated with increased recurrence risk (HR 1.82 per 5-mutation increase, p=0.004) (**Table 4**). These D2 tumors had significantly lower scores for cytotoxic immune features, such as CD8+ T cells (p=0.003), cytolytic activity (p<0.001), and IFN-γ response (p=0.001), and significantly higher scores for immunosuppressive elements, including Tregs (p=0.005), M2 macrophages (p=0.002), and a T-cell exhaustion signature (p=0.008). Similarly, B cell signature scores were significantly lower in the D2 subgroup compared to the D1 subgroup (median 1.05 [IQR 0.72–1.40] vs. 1.50 [IQR 1.15–1.95], p=0.012). (**Table 5** and **Figure 5**).

**Table 4:** Frequency of genomic alterations and association with DFS in the De-escalation cohort (N=65).

Gene	Alteration type	Overall frequency (N=65)	D1 (no recurrence, n=53)	D2 (recurrence, n=12)	Univariate cox HR (95% CI)	p
TP53	Mut/Trunc	46 (70.8%)	35 (66.0%)	11 (91.7%)	5.20 (1.15 – 23.58)	**0.032**
CDKN2A	Del/Mut	28 (43.1%)	19 (35.8%)	9 (75.0%)	3.87 (1.27 – 11.83)	**0.018**
NOTCH1	Mut/Trunc	22 (33.8%)	18 (34.0%)	4 (33.3%)	0.95 (0.30 – 3.04)	0.934
FAT1	Mut/Trunc	19 (29.2%)	15 (28.3%)	4 (33.3%)	1.25 (0.39 – 3.99)	0.703
PIK3CA	Mut/Ampl	17 (26.2%)	14 (26.4%)	3 (25.0%)	0.92 (0.26 – 3.30)	0.899
CASP8	Mut/Trunc	12 (18.5%)	10 (18.9%)	2 (16.7%)	0.86 (0.20 – 3.78)	0.844
HRAS	Mut	8 (12.3%)	7 (13.2%)	1 (8.3%)	0.61 (0.08 – 4.55)	0.629
B2M	Mut/Trunc	5 (7.7%)	3 (5.7%)	2 (16.7%)	2.78 (0.63 – 12.33)	0.180
JAK1	Mut/Trunc	4 (6.2%)	1 (1.9%)	3 (25.0%)	7.45 (2.07 – 26.78)	**0.002**
JAK2	Mut/Trunc	3 (4.6%)	1 (1.9%)	2 (16.7%)	8.12 (1.82 – 36.19)	**0.006**
STK11	Mut/Trunc	2 (3.1%)	1 (1.9%)	1 (8.3%)	4.20 (0.55 – 32.13)	0.165
BRCA1	Mut	3 (4.6%)	2 (3.8%)	1 (8.3%)	2.10 (0.27 – 16.18)	0.478
BRCA2	Mut	4 (6.2%)	3 (5.7%)	1 (8.3%)	1.45 (0.19 – 11.12)	0.722
ATM	Mut	6 (9.2%)	5 (9.4%)	1 (8.3%)	0.88 (0.12 – 6.65)	0.901
KMT2D	Mut/Trunc	14 (21.5%)	12 (22.6%)	2 (16.7%)	0.70 (0.16 – 3.06)	0.636
ARID1A	Mut/Trunc	9 (13.8%)	7 (13.2%)	2 (16.7%)	1.29 (0.29 – 5.68)	0.737
Any P53 pathway alteration*	Comp Alter	50 (76.9%)	39 (73.6%)	11 (91.7%)	4.01 (0.94 – 17.17)	0.061
Mutational Load	Per 5 mut increase	Median: 4 (IQR: 2-7)	4 (2-6)	7 (4-10)	1.82 (1.21 – 2.74)	**0.004**

Mut, mutation; Trunc, truncating; Del, homozygous deletion; Ampl, amplification; Comp Alter, composite alteration (mutation + copy number loss). *P53 pathway: TP53 mutation or CDKN2A deletion. Bold values means significant.

**Table 5:** Pre-treatment immune microenvironment scores and association with DFS in the De-escalation cohort.

Immune feature (nCounter Score)	D1 (no recurrence, n=53) median (IQR)	D2 (recurrence, n=12) median (IQR)	Mann-Whitney U	Univariate cox HR (95% CI)*
Cytotoxic cell infiltration				
CD8+ T cells	1.85 (1.42 – 2.30)	1.21 (0.85 – 1.55)	**0.003**	0.45 (0.26 – 0.78)
Cytolytic Activity Score	2.10 (1.65 – 2.58)	1.35 (0.95 – 1.80)	**<0.001**	0.38 (0.22 – 0.66)
Innate & adaptive immunity				
B cells	1.50 (1.15 – 1.95)	1.05 (0.72 – 1.40)	**0.012**	0.52 (0.30 – 0.89)
Dendritic Cells	1.65 (1.28 – 2.05)	1.30 (0.90 – 1.65)	0.051	0.60 (0.35 – 1.03)
NK Cells	1.40 (1.05 – 1.78)	1.10 (0.75 – 1.45)	0.078	0.64 (0.38 – 1.09)
Key functional pathways				
IFN-γ Response	2.25 (1.80 – 2.75)	1.60 (1.10 – 2.05)	**0.001**	0.42 (0.24 – 0.73)
Antigen Presentation Machinery	1.95 (1.55 – 2.40)	1.40 (1.00 – 1.85)	**0.007**	0.49 (0.28 – 0.85)
Checkpoint Molecules	1.70 (1.30 – 2.15)	1.25 (0.85 – 1.70)	**0.023**	0.55 (0.32 – 0.94)
Immunosuppressive features				
Tregs	0.90 (0.65 – 1.20)	1.40 (1.05 – 1.85)	**0.005**	2.85 (1.56 – 5.20)
M2 Macrophages	0.80 (0.55 – 1.10)	1.30 (0.95 – 1.70)	**0.002**	3.25 (1.75 – 6.04)
T-cell Exhaustion Signature	1.10 (0.80 – 1.45)	1.65 (1.25 – 2.10)	**0.008**	2.45 (1.40 – 4.29)

IQR, interquartile range; HR, Hazard Ratio; CI, Confidence Interval. *HRs are for a 1-unit decrease in protective immune scores (CD8, IFN-γ, etc.) and a 1-unit increase in immunosuppressive scores (Tregs, M2, etc.), derived from continuous Cox models. All listed HRs are for the direction of increased risk (HR >1). The corresponding p-values for these Cox models align with the significant Mann-Whitney U test results (p<0.05). Bold values means significant.

### Development of a clinical risk stratification tool

To translate these findings into a clinically actionable model, variables significant in univariate analysis were integrated into a multivariate Cox model followed by backward stepwise selection (**Table 6**). This process identified only two independent predictors of DFS: TP53 mutation status and the pre-treatment B cell signature score. In the final multivariable model, patients with a TP53 mutation faced a 4-fold increased risk of recurrence (adjusted HR 4.05, 95% CI 1.25–13.10, p=0.019), while each 1-unit decrease in the B cell score (indicating poorer B cell infiltration) was associated with a more than 2-fold increased risk (adjusted HR 2.15, 95% CI 1.20–3.85, p=0.010). Using median cutoffs for these biomarkers, a simple combinatorial rule classified patients into three distinct prognostic groups: Low-Risk (favorable TP53 WT and high B cell score), Intermediate-Risk (one unfavorable biomarker), and High-Risk (both unfavorable biomarkers). This stratification demonstrated powerful discriminatory power, with 0 recurrences among 15 Low-Risk patients, 6 recurrences among 35 Intermediate-Risk patients, and 6 recurrences among 15 High-Risk patients (**Figure 1**, p=0.019). The final two-biomarker model yielded a C-index of 0.74 (95% CI: 0.62–0.86). Bootstrap internal validation (1000 resamples) resulted in an optimism-corrected C-index of 0.71, suggesting moderate and stable discriminatory performance despite the modest sample size. The HRs for TP53 mutation (adjusted HR range across resamples: 3.8–4.3) and B-cell signature (adjusted HR range per 1-unit decrease: 2.0–2.3) remained consistent, supporting the robustness of these predictors within this exploratory cohort.

**Table 6:** Multivariate cox analysis for DFS in the de-escalation group (N=65).

Variable entered into model	Type	Univariate		Multivariate model 1 (Full)		Multivariate final model (Stepwise)	
		HR (95% CI)	P	Adjusted HR (95% CI)	P	Adjusted HR (95% CI)	P
*TP53*	Mutant vs. WT	5.20 (1.15 – 23.58)	**0.032**	3.85 (1.12 – 13.25)	**0.033**	4.05 (1.25 – 13.10)	**0.019**
*CDKN2A*	Altered vs. WT	3.87 (1.27 – 11.83)	**0.018**	1.95 (0.68 – 5.60)	0.212	–	–
*JAK1*	Mutant vs. WT	7.45 (2.07 – 26.78)	**0.002**	2.45 (0.75 – 8.01)	0.138	–	–
*JAK2*	Mutant vs. WT	8.12 (1.82 – 36.19)	**0.006**	2.88 (0.82 – 10.12)	0.099	–	–
Mutational Load	per 5-mut increase	1.82 (1.21 – 2.74)	**0.004**	1.25 (0.85 – 1.83)	0.256	–	–
CD8+ T Cell Score	per 1-unit decrease	2.22 (1.28 – 3.85)	**0.005**	1.42 (0.78 – 2.58)	0.250	–	–
B Cell Signature Score	per 1-unit decrease	1.92 (1.12 – 3.30)	**0.018**	2.05 (1.15 – 3.65)	**0.015**	2.15 (1.20 – 3.85)	**0.010**
IFN-γ Response Score	per 1-unit decrease	2.38 (1.37 – 4.12)	**0.002**	1.55 (0.82 – 2.94)	0.175	–	–
Treg Score	per 1-unit increase	2.85 (1.56 – 5.20)	**0.001**	1.65 (0.87 – 3.12)	0.125	–	–
M2 Macrophage Score	per 1-unit increase	3.25 (1.75 – 6.04)	**<0.001**	1.85 (0.92 – 3.72)	0.084	–	–
T-cell Exhaustion Score	per 1-unit increase	2.45 (1.40 – 4.29)	**0.002**	1.38 (0.72 – 2.65)	0.330	–	–

Model 1 (Full): All 11 variables with univariate p < 0.05 were entered into a single multivariate Cox model.

Variable Elimination: Backward stepwise selection (removal criterion: p > 0.05) was applied

Final Model: The procedure retained only two variables that maintained independent significance after adjusting for all others: *TP53* Mutation and B Cell Signature Score. Bold values means significant.

## Discussion

Our study demonstrates that for patients with locally advanced OSCC who achieve a pCR after NICT, the omission of adjuvant radiotherapy does not compromise disease control while conferring a substantial and sustained benefit to patient QoL. Crucially, our integrated biomarker analysis moves beyond the binary endpoint of pCR, revealing that the success of this de-escalation strategy is not uniform but is governed by the interplay of intrinsic tumor genomics and the pre-existing immune microenvironment. We distilled this biological complexity into a clinically actionable, two-biomarker model that can identify patients with near-zero recurrence risk, for whom de-escalation is highly suitable, and a high-risk subgroup for whom standard adjuvant radiotherapy may still be warranted.

In the evolving landscape of head and neck cancer management, treatment de-escalation has emerged as a pivotal strategy aimed at preserving oncologic efficacy while minimizing long-term morbidity and enhancing QoL. Traditional multimodal therapies often inflict substantial functional and cosmetic damage, leading to chronic issues such as xerostomia, dysphagia, and donor-site morbidity, which profoundly impair patients’ well-being (10). The recent success of perioperative immunotherapy has not only demonstrated improved event-free and DFS but also opened new avenues for response-adapted de-escalation (11). Notably, KEYNOTE-689 showed that neoadjuvant pembrolizumab reduced postoperative high-risk pathological features by approximately 11%, enabling lower cisplatin use and reduced radiotherapy doses (11). This aligns with the broader vision emphasizing that NICT can facilitate surgical downstaging, reduce radiation fields or intensity, and potentially spare patients from adjuvant chemotherapy altogether. Promising pathological response rate further support the feasibility of tailoring postoperative therapy to preoperative response (10). Treatment de-escalation in HPV-positive oropharyngeal squamous cell carcinoma is well supported with studies showing 5-year survival rates of 94.7%–100% using transoral surgery and risk-adapted, reduced-dose adjuvant therapy (12). In contrast, de-escalation in OSCC remains unproven, due to lacking favorable prognostic markers.

Recent advances in NICT have catalyzed a paradigm shift toward treatment de-escalation in OSCC, with several studies exploring response-adapted strategies to preserve oncologic outcomes while minimizing morbidity. Fang et al. (9) demonstrated that response-adapted surgery achieved equivalent 3-year event-free and overall survival compared to traditional radical surgery, while significantly reducing mandibulectomy rates, flap reconstructions, and major complications. Their findings align with our observation that aggressive adjuvant radiotherapy may be unnecessary in select patients who achieve a pCR, reinforcing the broader principle that robust treatment response can guide therapeutic moderation. Similarly, Wu et al. (12) and Cao et al. (13) reported high pCR rates after NICT and suggested that such responses correlate with favorable long-term outcomes, providing a rationale for postoperative therapy individualization. However, these studies retained radiotherapy as a core adjuvant modality. In contrast, we omit radiotherapy entirely and substitute it with sustained anti-PD-1 therapy, thereby shifting toxicity from chronic radiation sequelae to manageable immune-related adverse events. This modality replacement rather than dose reduction directly underpins the superior QoL observed in our cohort and represents a paradigm shift toward an immunotherapy-forward adjuvant strategy for biologically selected patients. While Fang et al. and others (9, 12, 13) relied on radiologic or major pathologic response, our cohort exclusively comprised patients with pCR. This allowed us to interrogate whether consolidative radiotherapy adds any survival benefit in the context of complete pathologic clearance.

Moreover, unlike previous reports that emphasized surgical or systemic de-intensification alone, our strategy replaces adjuvant radiotherapy with maintenance immunotherapy, thereby shifting toxicity profiles from chronic radiation sequelae to manageable immune-related adverse events. This trade-off resulted in significantly better QoL at 6 and 12 months, an advantage not fully captured in earlier surgical de-escalation studies that still administered standard adjuvant radiotherapy. The commentary by Liu et al. (14) on the feasibility of omitting adjuvant radiotherapy after neoadjuvant therapy and surgery in oral cavity cancer highlighted the need for personalized de-escalation based on pathological response. However, their analysis remained within the traditional framework of surgical and neoadjuvant optimization, without proposing an alternative adjuvant modality. Our approach advances this concept by actively substituting radiotherapy with sustained immunotherapy, thus addressing not only the question of whether to de-escalate, but how to do so in a way that rebalances efficacy and toxicity. This represents a paradigm shift from merely “omitting” a toxic component to “replacing” it with a mechanism-based, systemically active treatment that maintains antitumor immunity while avoiding localized tissue damage. Similarly, the UPGRADE-RT trial (15) demonstrated that reduced dose elective neck irradiation decreased acute dysphagia and tube feeding dependence. However, that study retained radiotherapy as the core adjuvant modality, focusing solely on dose reduction within the same treatment modality. In contrast, our strategy eliminates radiotherapy entirely for selected pCR patients, thereby not only reducing acute toxicity but also precluding the entire spectrum of chronic radiation. The trade-off we observed reflects a conscious modality substitution that aligns with the increasing durability and manageability of modern immunotherapy supportive care. The study by Ju et al. (16) found no survival difference between patients who did or did not receive adjuvant radiotherapy after neoadjuvant therapy and surgery, suggesting that radiotherapy might be omitted in certain subgroups. Nevertheless, that study did not specify what, if anything, should replace radiotherapy in the adjuvant setting, leaving a therapeutic vacuum that could increase recurrence anxiety. Our protocol fills this gap by implementing maintenance immunotherapy as the consolidative treatment, thereby providing continued systemic disease control while capitalizing on the immune. This creates a coherent “immunotherapy−forward” pathway from neoadjuvant to adjuvant treatment, which is biologically rational in the context of pCR.

Our risk stratification strategy integrating pretreatment tumor genomics with features of the immune microenvironment to guide de-escalation decisions in locally advanced OSCC is both biologically grounded and clinically innovative. The rationale draws strong support from converging lines of evidence across multiple recent studies. Emerging data highlight that OSCC is enriched for gain-of-function mutations in the NRF2 pathway and high-risk TP53 alterations, both of which are linked to aggressive biology and treatment resistance (17). Our approach extends beyond HPV status by incorporating specific genomic aberrations alongside immune contexture metrics, such as CD8+ T-cell infiltration and B-cell clonality. This dual-layered assessment aligns with findings (18, 19), which demonstrate that TP53-mutant tumors exhibit distinct immune landscapes and poorer survival, while co-mutation patterns can modulate immunogenicity and clinical outcomes.

Moreover, our model resonates with cutting-edge insights into the functional interplay between adaptive immunity and tumor genomics. TP53 dysfunction may foster an immunosuppressive microenvironment by impairing antigen presentation and recruiting suppressive immune cells, limiting the ability to clear residual disease after neoadjuvant therapy. Conversely, a robust B cell signature likely supports sustained anti-tumor immunity through tertiary lymphoid structure formation, enhanced T cell help, and cytokine secretion. Together, these biomarkers may identify tumors with inherent immune escape potential (20). Recent single-cell RNA sequencing studies (21, 22) have revealed that clonally expanded B cells and germinal center–like signatures correlate with CD8+ T-cell activation and improved survival in OSCC, suggesting that humoral immunity plays a non-redundant role in anti-tumor responses. By integrating such immune features into a combinatorial rule with genomic markers, our stratification system captures the multidimensional nature of treatment response more holistically than models based on pathology or genomics alone. This is particularly relevant in the post-neoadjuvant setting, where pCR may mask underlying heterogeneity in recurrence risk, as underscored by our observation that not all pCR patients fare equally well after radiotherapy omission.

The novelty of our framework lies in its prospective translation of integrated biomarker data into a simple, median-dichotomized, three-tier risk classification. Unlike complex machine-learning scores or continuous risk indices that lack clear clinical thresholds, our approach offers actionable cutoffs that can be implemented even in resource-limited settings. Furthermore, while prior efforts (17) have proposed biomarker panels for HPV+ oropharyngeal cancer, our focus on OSCC addresses an unmet clinical need. In sum, by anchoring risk prediction in both tumor-intrinsic and extrinsic determinants of therapeutic vulnerability, our model represents a significant advance toward precision de-escalation in a disease where overtreatment remains a major concern. While the TP53/B-cell model is clinically promising, its implementation faces practical challenges. TP53 mutation testing via NGS is widely available, but B-cell signature assessment is less routine and may require validation with simpler immunohistochemical markers. Cost, accessibility, and the need for prospective multicenter validation remain key hurdles before this biomarker strategy can be adopted into routine practice.

Our findings should be interpreted in light of several limitations. The retrospective design may leave residual confounding despite propensity score matching. The biomarker model was derived from a relatively small cohort of 65 patients with only 12 recurrence events. This limits the statistical power and increases the risk of overfitting, and thus these findings should be regarded as preliminary and hypothesis-generating. Although internal bootstrap validation suggested reasonable stability of the model, external validation in an independent, preferably prospective, cohort is essential before this two-biomarker stratification tool can be considered for clinical implementation. Future studies with larger sample sizes are also needed to explore additional genomic and immune variables that may further refine risk prediction in this setting. Our genomic analysis was restricted to a focused 18-gene panel; broader sequencing approaches may reveal additional predictive alterations.

In summary, our study demonstrates that omitting adjuvant radiotherapy in locally advanced OSCC patients who achieve pCR after NICT is an oncologically safe strategy that confers significant and sustained QoL benefits. By integrating pretreatment tumor genomics and immune microenvironment features, we developed a preliminary two-biomarker model that provides preliminary evidence for personalizing adjuvant therapy. This work is ideally positioned as a landmark exploratory study. Its core value lies in proposing a safe and feasible de-escalation strategy and a testable biomarker hypothesis, thereby paving the way for well-designed prospective phase II/III clinical trials in this field. The ultimate goal of this framework is to identify low-risk patients who might be candidates for radiotherapy de-escalation while preserving curability, minimizing treatment-related morbidity, and enhancing long-term patient well-being.

## Data Availability

The original contributions presented in the study are included in the article/**Supplementary Material**. Further inquiries can be directed to the corresponding author.
