# Supplementary material for: De-escalating radiotherapy in pathologic complete response oral cancer after neoadjuvant immunochemotherapy: equal survival, better life, and a biomarker guide
Source: Front Oncol. 2026 Feb 4;16:1761516. doi: 10.3389/fonc.2026.1761516 (PMC12913093; doi:10.3389/fonc.2026.1761516)
Supplement: Supplementary file 1 [file Table1.doc]

### ****Supplementary Table 1: Longitudinal Health-Related Quality of Life (EORTC QLQ-C30)****

| **Domain** | **Time point** | **De-escalation** | **Standard Care** | **p-value** |
| --- | --- | --- | --- | --- |
| Global QoL | Pre-adjuvant | 58.3 ± 12.5 | 56.0 ± 13.1 | 0.292 |
|  | 3 months-post | 65.1 ± 11.8 | 58.5 ± 12.9 | ****0.002**** |
|  | 6 months-post | 78.2 ± 10.1 | 66.8 ± 11.5 | ****<0.001**** |
|  | 12 months-post | 84.5 ± 8.3 | 75.2 ± 10.4 | ****<0.001**** |
| Physical functioning | Pre-adjuvant | 75.6 ± 14.2 | 73.8 ± 15.0 | 0.474 |
|  | 3 months-post | 72.4 ± 13.6 | 65.2 ± 14.8 | ****0.003**** |
|  | 6 months-post | 82.1 ± 11.2 | 70.5 ± 13.1 | ****<0.001**** |
|  | 12 months-post | 88.3 ± 9.1 | 78.9 ± 11.8 | ****<0.001**** |
| Role functioning | Pre-adjuvant | 70.2 ± 16.8 | 68.5 ± 17.2 | 0.569 |
|  | 3 months-post | 68.8 ± 15.1 | 58.9 ± 16.5 | ****<0.001**** |
|  | 6 months-post | 80.5 ± 12.4 | 67.2 ± 14.9 | ****<0.001**** |
|  | 12 months-post | 87.1 ± 10.5 | 76.4 ± 13.2 | ****<0.001**** |
| Emotional functioning | Pre-adjuvant | 68.9 ± 15.1 | 66.2 ± 16.0 | 0.324 |
|  | 3 months-post | 75.6 ± 12.9 | 70.1 ± 14.2 | ****0.017**** |
|  | 6 months-post | 81.3 ± 11.5 | 74.5 ± 13.8 | ****0.001**** |
|  | 12 months-post | 85.7 ± 9.8 | 79.8 ± 12.1 | ****0.002**** |
| Cognitive functioning | Pre-adjuvant | 82.4 ± 10.5 | 80.1 ± 11.3 | 0.224 |
|  | 3 months-post | 80.2 ± 11.1 | 76.5 ± 12.4 | 0.080 |
|  | 6 months-post | 85.6 ± 9.8 | 79.8 ± 11.6 | ****0.002**** |
|  | 12 months-post | 87.9 ± 8.7 | 82.1 ± 10.9 | ****0.001**** |
| Social functioning | Pre-adjuvant | 65.8 ± 17.5 | 63.4 ± 18.1 | 0.433 |
|  | 3 months-post | 70.5 ± 14.8 | 60.2 ± 16.9 | ****<0.001**** |
|  | 6 months-post | 83.4 ± 12.1 | 68.9 ± 15.4 | ****<0.001**** |
|  | 12 months-post | 89.2 ± 9.5 | 77.6 ± 13.7 | ****<0.001**** |
| Fatigue | Pre-adjuvant | 45.6 ± 18.2 | 48.2 ± 19.1 | 0.414 |
|  | 3 months-post | 50.2 ± 16.5 | 58.9 ± 17.8 | ****0.004**** |
|  | 6 months-post | 35.8 ± 14.1 | 45.6 ± 16.2 | ****<0.001**** |
|  | 12 months-post | 28.4 ± 12.3 | 36.7 ± 14.9 | ****<0.001**** |
| Nausea/vomiting | Pre-adjuvant | 12.3 ± 8.5 | 14.1 ± 9.2 | 0.223 |
|  | 3 months-post | 25.6 ± 10.2 | 30.5 ± 11.5 | ****0.012**** |
|  | 6 months-post | 10.2 ± 7.1 | 18.7 ± 9.8 | ****<0.001**** |
|  | 12 months-post | 6.8 ± 5.9 | 12.3 ± 8.4 | ****<0.001**** |
| Pain | Pre-adjuvant | 40.2 ± 16.8 | 42.5 ± 17.5 | 0.424 |
|  | 3 months-post | 35.6 ± 14.2 | 50.8 ± 16.1 | ****<0.001**** |
|  | 6 months-post | 22.1 ± 11.5 | 35.4 ± 14.8 | ****<0.001**** |
|  | 12 months-post | 15.3 ± 9.8 | 24.6 ± 12.1 | ****<0.001**** |
| Dyspnea | Pre-adjuvant | 15.4 ± 9.8 | 16.8 ± 10.5 | 0.434 |
|  | 3 months-post | 18.9 ± 8.7 | 22.1 ± 9.9 | ****0.046**** |
|  | 6 months-post | 12.3 ± 7.4 | 18.5 ± 9.1 | ****<0.001**** |
|  | 12 months-post | 10.1 ± 6.5 | 15.4 ± 8.2 | ****<0.001**** |
| Insomnia | Pre-adjuvant | 35.6 ± 14.9 | 38.9 ± 15.7 | 0.224 |
|  | 3 months-post | 40.2 ± 13.5 | 48.7 ± 14.8 | ****<0.001**** |
|  | 6 months-post | 25.6 ± 11.2 | 35.2 ± 13.6 | ****<0.001**** |
|  | 12 months-post | 18.9 ± 9.8 | 26.8 ± 12.1 | ****<0.001**** |
| Appetite loss | Pre-adjuvant | 30.5 ± 12.5 | 32.1 ± 13.3 | 0.469 |
|  | 3 months-post | 38.9 ± 11.2 | 52.3 ± 12.9 | ****<0.001**** |
|  | 6 months-post | 20.1 ± 9.8 | 35.6 ± 11.7 | ****<0.001**** |
|  | 12 months-post | 12.4 ± 8.1 | 22.1 ± 10.5 | ****<0.001**** |
| Constipation | Pre-adjuvant | 18.9 ± 10.1 | 20.2 ± 10.8 | 0.492 |
|  | 3 months-post | 22.1 ± 9.5 | 28.7 ± 10.9 | ****<0.001**** |
|  | 6 months-post | 15.4 ± 8.2 | 22.3 ± 9.8 | ****<0.001**** |
|  | 12 months-post | 10.5 ± 7.1 | 16.8 ± 8.9 | ****<0.001**** |
| Diarrhea | Pre-adjuvant | 10.2 ± 6.8 | 11.5 ± 7.4 | 0.287 |
|  | 3 months-post | 12.8 ± 6.1 | 15.6 ± 7.2 | ****0.018**** |
|  | 6 months-post | 9.1 ± 5.5 | 13.4 ± 6.9 | ****<0.001**** |
|  | 12 months-post | 7.8 ± 4.9 | 11.2 ± 6.3 | ****<0.001**** |
| Financial difficulty | Pre-adjuvant | 25.6 ± 11.2 | 28.9 ± 12.0 | 0.095 |
|  | 3 months-post | 35.8 ± 10.5 | 45.6 ± 11.9 | ****<0.001**** |
|  | 6 months-post | 28.9 ± 9.1 | 38.7 ± 10.8 | ****<0.001**** |
|  | 12 months-post | 20.1 ± 8.2 | 30.5 ± 10.1 | ****<0.001**** |

****Scoring Note:**** All scores are presented as mean ± standard deviation. For functioning and Global QoL scales, a higher score represents a better level of functioning or QoL. For symptom scales, a higher score represents a greater severity of symptoms.
