# Supplementary material for: De-escalating radiotherapy in pathologic complete response oral cancer after neoadjuvant immunochemotherapy: equal survival, better life, and a biomarker guide
Source: Front Oncol. 2026 Feb 4;16:1761516. doi: 10.3389/fonc.2026.1761516 (PMC12913093; doi:10.3389/fonc.2026.1761516)
Supplement: Supplementary file 2 [file Table2.doc]

### ****Supplementary Table 2: Longitudinal Head and Neck-Specific Quality of Life (EORTC QLQ-H&N35)****

| **Domain** | **Time point** | **De-escalation** | **Standard Care** | **p-value** |
| --- | --- | --- | --- | --- |
| Pain | Pre-adjuvant | 45.2 ± 16.5 | 47.8 ± 17.2 | 0.382 |
|  | 3 months-post | 38.5 ± 14.1 | 52.3 ± 16.8 | ****<0.001**** |
|  | 6 months-post | 25.6 ± 11.8 | 38.9 ± 15.1 | ****<0.001**** |
|  | 12 months-post | 18.9 ± 9.5 | 28.7 ± 12.4 | ****<0.001**** |
| Swallowing | Pre-adjuvant | 35.8 ± 14.9 | 38.2 ± 15.7 | 0.382 |
|  | 3 months-post | 42.1 ± 13.5 | 55.6 ± 16.2 | ****<0.001**** |
|  | 6 months-post | 28.9 ± 11.2 | 42.3 ± 14.8 | ****<0.001**** |
|  | 12 months-post | 20.5 ± 9.1 | 32.1 ± 12.6 | ****<0.001**** |
| Senses | Pre-adjuvant | 25.6 ± 12.1 | 27.8 ± 12.9 | 0.329 |
|  | 3 months-post | 28.9 ± 11.5 | 32.1 ± 13.2 | 0.148 |
|  | 6 months-post | 22.1 ± 9.8 | 28.7 ± 12.1 | ****<0.001**** |
|  | 12 months-post | 18.9 ± 8.4 | 24.5 ± 10.8 | ****0.001**** |
| Speech | Pre-adjuvant | 32.1 ± 13.8 | 34.5 ± 14.6 | 0.369 |
|  | 3 months-post | 35.6 ± 12.4 | 48.9 ± 15.1 | ****<0.001**** |
|  | 6 months-post | 22.3 ± 10.5 | 35.2 ± 13.9 | ****<0.001**** |
|  | 12 months-post | 15.8 ± 8.7 | 26.8 ± 12.1 | ****<0.001**** |
| Social eating | Pre-adjuvant | 40.2 ± 15.8 | 42.9 ± 16.5 | 0.355 |
|  | 3 months-post | 45.6 ± 14.2 | 58.7 ± 17.1 | ****<0.001**** |
|  | 6 months-post | 30.5 ± 12.1 | 45.1 ± 15.8 | ****<0.001**** |
|  | 12 months-post | 22.1 ± 10.2 | 35.6 ± 14.5 | ****<0.001**** |
| Social contact | Pre-adjuvant | 28.9 ± 11.5 | 30.5 ± 12.3 | 0.469 |
|  | 3 months-post | 32.1 ± 10.8 | 42.3 ± 13.5 | ****<0.001**** |
|  | 6 months-post | 20.8 ± 9.1 | 32.6 ± 12.4 | ****<0.001**** |
|  | 12 months-post | 15.6 ± 7.8 | 25.4 ± 11.2 | ****<0.001**** |
| Sexual problems | Pre-adjuvant | 52.3 ± 18.2 | 54.1 ± 19.0 | 0.593 |
|  | 3 months-post | 55.6 ± 16.8 | 58.9 ± 18.1 | 0.289 |
|  | 6 months-post | 48.9 ± 15.1 | 52.3 ± 17.2 | 0.246 |
|  | 12 months-post | 45.2 ± 13.8 | 48.7 ± 16.5 | 0.214 |
| Teeth | Pre-adjuvant | 38.7 ± 14.5 | 40.2 ± 15.3 | 0.582 |
|  | 3 months-post | 42.1 ± 13.2 | 55.8 ± 16.1 | ****<0.001**** |
|  | 6 months-post | 30.5 ± 11.5 | 45.6 ± 14.9 | ****<0.001**** |
|  | 12 months-post | 25.6 ± 10.1 | 38.9 ± 13.8 | ****<0.001**** |
| Open mouth | Pre-adjuvant | 35.6 ± 13.9 | 37.8 ± 14.7 | 0.403 |
|  | 3 months-post | 40.2 ± 12.5 | 52.3 ± 15.8 | ****<0.001**** |
|  | 6 months-post | 28.9 ± 10.8 | 42.1 ± 14.2 | ****<0.001**** |
|  | 12 months-post | 22.1 ± 9.5 | 35.6 ± 12.9 | ****<0.001**** |
| Dry mouth | Pre-adjuvant | 48.9 ± 16.8 | 50.5 ± 17.6 | 0.606 |
|  | 3 months-post | 52.3 ± 15.5 | 58.7 ± 17.2 | ****0.026**** |
|  | 6 months-post | 45.6 ± 13.9 | 52.1 ± 16.1 | ****0.015**** |
|  | 12 months-post | 40.2 ± 12.5 | 48.9 ± 15.8 | ****<0.001**** |
| Sticky saliva | Pre-adjuvant | 42.1 ± 15.2 | 44.5 ± 16.0 | 0.380 |
|  | 3 months-post | 45.6 ± 14.1 | 52.3 ± 16.5 | ****0.012**** |
|  | 6 months-post | 38.9 ± 12.8 | 48.7 ± 15.9 | ****<0.001**** |
|  | 12 months-post | 35.2 ± 11.5 | 42.1 ± 14.2 | ****0.002**** |
| Cough | Pre-adjuvant | 28.7 ± 11.8 | 30.2 ± 12.6 | 0.498 |
|  | 3 months-post | 32.1 ± 10.9 | 35.6 ± 13.2 | 0.106 |
|  | 6 months-post | 25.6 ± 9.8 | 30.5 ± 12.1 | ****0.016**** |
|  | 12 months-post | 22.1 ± 8.5 | 28.7 ± 11.4 | ****<0.001**** |
| Feeling ill | Pre-adjuvant | 35.2 ± 13.5 | 37.8 ± 14.3 | 0.297 |
|  | 3 months-post | 38.9 ± 12.8 | 45.6 ± 15.1 | ****0.009**** |
|  | 6 months-post | 28.7 ± 11.2 | 38.9 ± 14.5 | ****<0.001**** |
|  | 12 months-post | 22.3 ± 9.8 | 32.1 ± 13.2 | ****<0.001**** |
| Pain medication | Pre-adjuvant | 22.1 ± 9.8 | 24.5 ± 10.5 | 0.188 |
|  | 3 months-post | 35.6 ± 11.2 | 48.9 ± 13.8 | ****<0.001**** |
|  | 6 months-post | 18.9 ± 8.5 | 32.1 ± 12.1 | ****<0.001**** |
|  | 12 months-post | 12.4 ± 7.1 | 25.6 ± 10.9 | ****<0.001**** |
| Food supplement | Pre-adjuvant | 18.9 ± 8.7 | 20.2 ± 9.4 | 0.414 |
|  | 3 months-post | 32.1 ± 10.5 | 45.6 ± 12.9 | ****<0.001**** |
|  | 6 months-post | 15.6 ± 7.8 | 28.9 ± 11.5 | ****<0.001**** |
|  | 12 months-post | 10.2 ± 6.5 | 22.1 ± 10.2 | ****<0.001**** |
| Feeding tube | Pre-adjuvant | 15.6 ± 7.5 | 16.8 ± 8.2 | 0.408 |
|  | 3 months-post | 28.9 ± 9.8 | 42.1 ± 12.1 | ****<0.001**** |
|  | 6 months-post | 12.3 ± 6.8 | 25.6 ± 10.5 | ****<0.001**** |
|  | 12 months-post | 8.9 ± 5.9 | 18.9 ± 9.8 | ****<0.001**** |
| Weight loss | Pre-adjuvant | 25.6 ± 10.8 | 27.8 ± 11.6 | 0.277 |
|  | 3 months-post | 35.2 ± 11.5 | 48.7 ± 14.2 | ****<0.001**** |
|  | 6 months-post | 20.1 ± 9.1 | 35.6 ± 12.8 | ****<0.001**** |
|  | 12 months-post | 15.6 ± 7.8 | 28.9 ± 11.5 | ****<0.001**** |
| Weight gain | Pre-adjuvant | 12.3 ± 6.8 | 13.5 ± 7.4 | 0.351 |
|  | 3 months-post | 15.6 ± 6.2 | 18.9 ± 8.1 | ****0.011**** |
|  | 6 months-post | 18.9 ± 7.1 | 22.1 ± 9.2 | ****0.035**** |
|  | 12 months-post | 22.1 ± 8.5 | 25.6 ± 10.1 | ****0.037**** |

****Scoring Note:**** All scores are presented as mean ± standard deviation. For all symptom scales, a higher score represents a greater severity of symptoms.
